# Comparison of waist to height ratio and body indices for prediction of metabolic disturbances in the Korean population: the Korean National Health and Nutrition Examination Survey 2008–2011

**DOI:** 10.1186/s12902-015-0075-5

**Published:** 2015-12-08

**Authors:** Seok Hui Kang, Kyu Hyang Cho, Jong Won Park, Jun Young Do

**Affiliations:** Division of Nephrology, Department of Internal Medicine, Yeungnam University Hospital, 317-1 Daemyung-Dong, Nam-Ku, Daegu 705-717 South Korea

**Keywords:** Waist to height ratio, Body composition, Metabolic syndrome, Insulin resistance

## Abstract

**Background:**

The aim of the present study of the general population was to identify the best predictor of metabolic risk among the body index variables evaluated with dual-energy X-ray absorptiometry (DEXA) or anthropometric indices including the waist to height ratio (WHtR).

**Patients and Methods:**

Data from the Korean National Health and Nutrition Examination Survey 2008**–**2011 were used for the analyses. As a result, 15,965 participants were included in this study. The body mass (BM) index was calculated as the body weight divided by the height squared. The WHtR was calculated as the waist circumference divided by height. Body composition indices such as lean mass (LM), fat mass (FM), trunk fat mass (TFM), and bone mineral content (BMC) were determined by using DEXA. Skeletal muscle mass (SM) was defined as the sum of the lean soft masses of both extremities. The LM, FM, BMC, TFM, and SM indices were calculated by dividing the total LM, total FM, total BMC, TFM, or SM by the height squared.

**Results:**

The WHtR had the highest area under the curve (AUC) and was the best predictor of metabolic syndrome for both sexes. In addition, the WHtR had the highest AUCs for components of metabolic syndrome (male: AUC 0.823, 95 % confidence interval [CI] 0.814**–**0.832; female: AUC 0.870, 95 % CI 0.863**–**0.877). There was a small statistically significant difference in AUC between WHtR and the other indices. Multivariate logistic regression showed that male participants in the second, third, and fourth quartiles had a 4.0 (95 % CI, 3.1**–**5.2), 9.6 (95 % CI, 7.5**–**12.3), and 36.1 (95 % CI, 28.0**–**46.4) times increased risk of metabolic syndrome compared with patients in the first quartile and female participants in the second, third, and fourth quartiles had a 4.3 (95 % CI, 3.1**–**6.0), 18.0 (95 % CI, 13.3**–**24.5), and 58.5 (95 % CI, 42.9**–**79.9) times increased risk of metabolic syndrome compared with patients in the first quartile.

**Conclusion:**

Among the BM, FM, LM, SM, TFM, and WHtR indices, WHtR is most useful to predict the presence of metabolic syndrome and insulin resistance in the Korean population.

**Electronic supplementary material:**

The online version of this article (doi:10.1186/s12902-015-0075-5) contains supplementary material, which is available to authorized users.

## Background

Metabolic syndrome is a pathologic condition characterized by elevated waist circumference (WC), elevated triglyceride level, reduced high-density lipoprotein cholesterol level, elevated blood pressure, and elevated fasting glucose level [1]. Metabolic syndrome is a risk factor for type 2 diabetes mellitus, and cardiovascular disease is a common health problem in patients with this condition; as a result, metabolic syndrome is associated with a risk of death and complications caused by cardiovascular disease and diabetes mellitus [2, 3]. Early detection and proper management of metabolic syndrome are important for prevention of cardiometabolic problems [4, 5].

Metabolic syndrome is closely related to obesity. Body mass index (BMI) and WC are well-known indices of general and abdominal obesity, respectively, and WC is a component of metabolic syndrome. However, various studies have reported that the waist to height ratio (WHtR), which considers ethnicity and height is more closely associated with insulin resistance and clinical outcomes in both adults and children [6–9].

Classic body indices such as BMI, WC, and WHtR, have been used to assess metabolic problems and have several advantages as simple rapid screening tools. However, they do not provide the distribution between skeletal muscle mass and fat. Body composition analyzers such as multi-frequency bioimpedance analysis and dual-energy X-ray absorptiometry (DEXA), can differentiate between muscle mass and fat and the distribution between limb and trunk.

Recent studies using body composition analyzers have reported that fat mass (FM) is positively correlated with metabolic syndrome [10–16]. However, some large-scale studies did not show that indices using body composition analyzers are superior to classic indices [17–19]. The aim of the present study of the general population was to identify the best predictor of metabolic risk among the body index variables evaluated with DEXA or anthropometric indices including the WHtR.

## Patients and Methods

### Study population

Data from the Korean National Health and Nutrition Examination Survey (KNHANES) 2008–2011 were used for the analyses. The KNHANES is a nationwide, multi-stage stratified survey of a representative sample of the entire South Korean population conducted by the Korea Centers for Disease Control and Prevention. The total number of participants in KNHANES was 37,753. Those who had no information regarding renal function and/or metabolic markers (*n* = 14,533), had no information regarding body composition (*n* = 6,019), or were younger than 18 years of age (*n* = 1,236) were excluded. As a result, 15,965 participants were included in this study. This study was approved by the institutional review board of Yeungnam University Hospital (YUH-14-0454-O56). The board waived the need for informed consent.

### Study variables

Clinical and laboratory data collected during the health examination included age, sex, education level, smoking behavior, mean daily alcohol intake, physical activity, WC (cm), WHtR, BMI (kg/m^2^), lean mass (LM) index (kg/m^2^), FM index (kg/m^2^), trunk FM (TFM) index (kg/m^2^), skeletal muscle mass (SM) index (kg/m^2^), bone mineral content (BMC) index (kg/m^2^), systolic blood pressure (mmHg), diastolic blood pressure (mmHg), fasting glucose level (mg/dL), total cholesterol level (mg/dL), triglyceride level (mg/dL), high-density lipoprotein (mg/dL), and estimated glomerular filtration rate (eGFR) (mL/min/1.73 m^2^).

Fasting glucose, total cholesterol, triglyceride, high-density lipoprotein levels were measured by a Hitachi Automatic Analyzer 7600 (Hitachi, Japan) by the enzymatic method (Sekisui Co., Japan). Serum creatinine level was measured by using a Hitachi Automatic Analyzer (alkaline picrate, Jaffé kinetic). The eGFR was calculated using the Chronic Kidney Disease Epidemiology Collaboration equation [20]. Chronic kidney disease was defined as an eGFR <60 mL/min/1.73 m^2^ or dipstick proteinuria (≥1+). Physical activity was assessed by self-reported questionnaires that examined frequency, intensity, and time per day spent on physical activity. We calculated metabolic equivalent-minutes per week (MET min/wk) using a physical activity calculating International Physical Activity Questionnaire and divided into 3 groups (<3000 MET min/wk, 3000–6000 MET min/wk, and ≥6000 MET min/wk) [21]. We defined smoking status in 2 respects: “smoking status” and “smoking amount” (PYR, average daily smoking amount [pack] × smoking period [year]). The cohorts were divided into 4 groups accordingly: heavy smoker (current smoker, ≥30 PYR), intermediate smoker (current smoker, <30 PYR), ex-smoker, and non-smoker [22]. Mean daily alcohol intake was defined as the Korean version of a “standard drinking”, which was based on WHO classification [23, 24]. We classified mean daily alcohol intake into 3 categories: abstinence (not having had a drink containing alcohol within the last year), moderate drinking (women, 0.1–19.99 g pure alcohol/day; men, 0.1–39.99 g pure alcohol/day), and heavy drinking (women, ≥20 g pure alcohol/day; men, ≥40 g pure alcohol/day). Weight and height were measured by well-trained medical professionals. Standing height was measured with the subject facing directly ahead with shoes off, feet together, arms by the sides, and heels, buttocks, and upper back in contact with the wall using a SECA 225 (SECA, Hamburg, Germany). Weight was measured using a GL-6000-20 scale (Cass, Seoul, Korea). WC was measured at the midpoint between the bottom of the rib cage and the top of the iliac crest. WHtR was calculated as WC divided by height. Body composition measurements such as LM, FM, and BMC, were determined by using DEXA (QDR 4500A; Hologic Inc., Waltham, MA, USA). SM was defined as the sum of the lean soft masses of both extremities. The LM, FM, BMC, TFM, and SM indices were calculated by dividing the total LM, total FM, total BMC, TFM, or SM by the height squared (kg/m^2^).

Metabolic syndrome was defined by the current criteria in the National Cholesterol Education Program Adult Treatment Panel III guidelines [1]. Elevated blood pressure was defined as a systolic or diastolic blood pressure of ≥130/85 mmHg, a self-reported history of hypertension, or the use of anti-hypertensive drugs. Elevated blood glucose was defined as a fasting blood glucose level ≥100 mg/dL or a self-reported history of diabetes mellitus. A low high-density lipoprotein cholesterol level was defined as <40 mg/dL in men and <50 mg/dL in women. Elevated triglyceride was defined as a serum triglyceride ≥150 mg/dL. Abdominal obesity was defined as a WC >90 cm in men and >80 cm in women. Metabolic syndrome was defined as the presence of ≥3 components of metabolic syndrome.

### Statistical analyses

The data were analyzed by SPSS version 19 (SPSS, Chicago, IL, USA). The distribution of continuous variables was checked by using the Kolmogorov–Smirnov test. Non-parametric variables were expressed as median (interquartile range) and compared by using the Mann–Whitney U–test. Categorical variables were expressed as counts and percentages. Pearson χ^2^ test or Fisher exact test was used to analyze categorical variables. Multivariate logistic regression was used to estimate the odds ratios, and the 95 % confidence intervals were used to determine the relationship between variables and metabolic syndrome. Covariates that were considered potential confounders (age, smoking, physical activity, and mean daily alcohol intake) were included in multivariate models. Model 1 was unadjusted, model 2 was adjusted for age, smoking, physical activity, and mean daily alcohol intake. Metabolic syndrome components were analyzed using analysis of covariance. Discrimination, which is the ability of the model to differentiate between participants who have metabolic syndrome and those who do not, was examined using the area under the receiver operating characteristic curve (AUROC). AUROC analysis was also performed to calculate cutoff values, sensitivity, and specificity. The best cutoff risk point was defined from the maximum of the Youden index in the AUROC. The statistical significance between the areas under indices receiver operating characteristic curves was calculated by the DeLong method. The AUROC was calculated by using MedCalc version 11.6.1.0 (MedCalc, Mariakerke, Belgium). The level of statistical significance was set at *P* < 0.05.

## Results

### Baseline characteristics of participants

The mean age of the study participants was 48 (36–62) years for men (*n* = 6875) and 48 (36–61) years for women (*n* = 9090) (Table 1). The number of participants with education levels of up to elementary school, middle school, high school, and college or higher was 1,263 (18.4 %), 871 (12.7 %), 2,471 (35.9 %), and 2,231 (32.5 %), respectively, in men, and 3,033 (33.4 %), 965 (10.6 %), 2,975 (32.7 %), and 2,070 (22.8 %), respectively, in women (*P* < 0.001). The education level was higher in men than in women. The male participants had higher body indices, including WC, WHtR, BMI, LM index, BMC index, SM index, and TFM index, than the female participants. The male participants also had significantly higher blood pressure, fasting glucose levels, triglyceride levels, and lower levels of high-density lipoprotein cholesterol than the female participants. In addition, the male participants had a higher prevalence of heavy drinking, current smoking, and chronic kidney disease than the female participants.

**Table 1:** Clinical characteristics of the participants

Characteristics	Males (*n* = 6875)	Females (*n* = 9090)	*P* value*
Age	48 (36–62)	48 (36–61)	0.748
WC (cm)	84.2 (78.2–90.2)	77.8 (71.1–85.0)	<0.001
WHtR	0.497 (0.459–0.533)	0.497 (0.450–0.548)	<0.001
BMI (kg/m^2^)	23.9 (21.8–25.9)	23.0 (20.9–25.4)	<0.001
LM index (kg/m^2^)	17.4 (16.3–18.6)	14.5 (13.4–15.6)	<0.001
FM index (kg/m^2^)	5.3 (4.0–6.5)	7.6 (6.2–9.1)	<0.001
BMC index (kg/m^2^)	0.88 (0.81–0.95)	0.80 (0.71–0.88)	<0.001
SM index (kg/m^2^)	7.6 (7.1–8.2)	5.8 (5.4–6.3)	<0.001
TFM index (kg/m^2^)	8.7 (8.1–9.3)	7.5 (6.9–8.1)	<0.001
SBP (mmHg)	116.5 (107.5–128.5)	111.9 (101.9–126.9)	<0.001
DBP (mmHg)	76.5 (69.3–84.5)	71.9 (66.9–78.9)	<0.001
Fasting glucose (mg/dL)	94 (88–103)	92 (86–99)	<0.001
Total cholesterol (mg/dL)	183 (161–208)	185 (163–210)	<0.001
HDL cholesterol (mg/dL)	48 (41–56)	53 (45–62)	<0.001
Triglyceride (mg/dL)	123 (84–187)	94 (65–140)	<0.001
Smoking			<0.001
Heavy smoker	761 (11.1 %)	28 (0.3 %)	
Intermediate smoker	3645 (53.0 %)	778 (8.6 %)	
Ex-smoker	1059 (15.4 %)	185 (2.0 %)	
Non-smoker	1279 (18.6 %)	7935 (87.3 %)	
No data	42 (0.6 %)	68 (0.7 %)	
Mean daily alcohol intake			<0.001
Abstinence	1127 (16.4 %)	3387 (37.3 %)	
Moderate drinking	4107 (59.7 %)	5138 (56.5 %)	
Heavy drinking	1591 (23.1 %)	494 (5.4 %)	
No data	50 (0.7 %)	71 (0.8 %)	
Exercise			<0.001
≥ 6000 MET min/wk	1666 (24.2 %)	1479 (16.3 %)	
3000 ~ 6000 MET min/wk	1466 (21.3 %)	1456 (16.0 %)	
<3000 MET min/wk	3672 (53.4 %)	6039 (66.4 %)	
No data	71 (1.0 %)	116 (1.3 %)	
Metabolic syndrome	2159 (31.4 %)	2932 (32.3 %)	0.258
eGFR (mL/min/1.73 m^2^)	93.7 (83.2–104.6)	101.3 (89.0–113.9)	<0.001
CKD	307 (4.5 %)	283 (3.1 %)	<0.001

Data are expressed as numbers (percentages) for categorical variables and median (interquartile range) for continuous variables

*Abbreviations*: *WC* waist circumference; *WHtR* waist to height ratio; *BMI* body mass index; *LM* lean mass; *FM* fat mass; *BMC* bone mineral content; *SM* skeletal muscle mass; *TFM* trunk fat mass; *SBP* systolic blood pressure; *DBP* diastolic blood pressure; *HDL* high-density lipoprotein; *MET min/wk* metabolic equivalent-minutes per week; *eGFR* estimated glomerular filtration rate; *CKD* chronic kidney disease

*Statistical significance was tested by using the Mann–Whitney U–test for continuous variables and Pearson χ^2^ test or Fisher exact test for categorical variables

### Comparison of the prediction of metabolic syndrome among the body indices

The body indices for prediction of metabolic syndrome were explored using AUROC curves, as shown in Additional file 1: Table S2. The WHtR had the highest area under the curve (AUC) (male: 0.823, 95 % CI 0.814–0.832; female: 0.870, 95 % CI 0.863–0.877) and was the best predictor of metabolic syndrome for both sexes. The sensitivity of predicting metabolic syndrome by WHtR was 74.0 % for the male participants and 83.3 % for the female participants, and the specificity was 75.8 % for the male participants and 76.5 % for the female participants. Considering the indices with an AUC < 0.6 to be poor measures, the BMC index had the lowest AUC (<0.6) and was poorest measure in predicting metabolic syndrome in both sexes.

In addition, the WHtR had the highest AUCs for components of metabolic syndrome, including elevated fasting glucose level, elevated blood pressure, elevated triglyceride level, and reduced high-density lipoprotein cholesterol level. In males, the SM and BMC indices were poor measures in predicting all metabolic syndrome components (Table 2). The LM index was predictive for only elevated triglyceride level among the metabolic syndrome components. In females, the BMC index was a poor measure in predicting all metabolic syndrome components. The SM index was predictive for only elevated fasting glucose and blood pressure levels among the metabolic syndrome components.

**Table 2:** The AUROC of each of the indices for the presence of metabolic syndrome components

Variables	Elevated FG	Elevated BP	Elevated TG	Decreased HDL-C
Males	AUC (95 % CI)	AUC (95 % CI)	AUC (95 % CI)	AUC (95 % CI)
BMI	0.611 (0.600**–**0.623)^*^	0.615 (0.603**–**0.627)^*^	0.672 (0.660**–**0.683)^*^	0.634 (0.622**–**0.645)^*^
FM index	0.613 (0.601**–**0.624)^*^	0.625 (0.614**–**0.637)^*^	0.679 (0.668**–**0.690)^**^	0.642 (0.630**–**0.653)^**^
LM index	0.571 (0.560**–**0.583)^*^	0.569 (0.557**–**0.581)^*^	0.611 (0.599**–**0.623)^*^	0.587 (0.575**–**0.599)^*^
BMC index	0.536 (0.525**–**0.548)^*^	0.517 (0.505**–**0.529)^*^	0.531 (0.519**–**0.543)^*^	0.540 (0.528**–**0.552)^*^
SM index	0.505 (0.493**–**0.517)^*^	0.506 (0.494**–**0.518)^*^	0.572 (0.561**–**0.584)^*^	0.546 (0.534**–**0.558)^*^
TFM index	0.620 (0.609**–**0.632)^*^	0.615 (0.604**–**0.627)^*^	0.631 (0.619**–**0.642)^*^	0.614 (0.602**–**0.625)^*^
WHtR	0.685 (0.674**–**0.696)	0.677 (0.665**–**0.688)	0.693 (0.682**–**0.704)	0.657 (0.644**–**0.667)
Females	AUC (95 % CI)	AUC (95 % CI)	AUC (95 % CI)	AUC (95 % CI)
BMI	0.689 (0.679**–**0.698)^*^	0.693 (0.683**–**0.703)^*^	0.688 (0.678**–**0.698)^*^	0.640 (0.630**–**0.650)^*^
FM index	0.665 (0.655**–**0.674)^*^	0.676 (0.666**–**0.686)^*^	0.680 (0.670**–**0.689)^*^	0.627 (0.617**–**0.637)^*^
LM index	0.670 (0.660**–**0.680)^*^	0.670 (0.660**–**0.679)^*^	0.650 (0.640**–**0.660)^*^	0.621 (0.611**–**0.631)^*^
BMC index	0.550 (0.540**–**0.560)^*^	0.625 (0.615**–**0.635)^*^	0.570 (0.559**–**0.580)^*^	0.534 (0.524**–**0.544)^*^
SM index	0.615 (0.605**–**0.625)^*^	0.604 (0.594**–**0.614)^*^	0.597 (0.587**–**0.607)^*^	0.595 (0.585**–**0.605)^*^
TFM index	0.690 (0.680**–**0.699)^*^	0.684 (0.674**–**0.693)^*^	0.664 (0.654**–**0.674)^*^	0.621 (0.611**–**0.631)^*^
WHtR	0.735 (0.726**–**0.744)	0.776 (0.768**–**0.785)	0.738 (0.729**–**0.747)	0.671 (0.662**–**0.681)

*Abbreviations*: *AUROC* area under the receiver operating characteristic curve; *FG* fasting glucose; *BP* blood pressure; *TG* triglyceride; *HDL-C* high density lipoprotein cholesterol; *AUC* area under the curve; *CI* confidence interval; *BMI*, body mass index; *FM* fat mass; *LM* lean mass; *BMC* bone mineral content; *SM* skeletal muscle mass; *TFM* trunk fat mass; *WHtR* waist to height ratio

^*^ *P* < 0.001 compared with the WHtR

^**^ *P* <0.01 compared with the WHtR

### Presence of metabolic syndrome according to WHtR quartiles

The WHtRs were divided into 4 quartiles. The quartiles for male participants were as follows: first quartile (Q1), ≤0.4603; second quartile (Q2), 0.4604–0.4981; third quartile (Q3), 0.4982–0.5343; and fourth quartile (Q4), ≥0.5344. The quartiles for the female participants were as follows: Q1, ≤0.4520; Q2, 0.4521–0.5002; Q3, 0.5003–0.5510; and Q4, ≥0.5511. In model 2, male participants in Q2, Q3, and Q4 had a 4.0 (95 % CI 3.1–5.2), 9.6 (95 % CI 7.5–12.3), and 36.1 (95 % CI 28.0–46.4) times increased risk of metabolic syndrome compared with patients in Q1 and female participants in the Q2, Q3, and Q4 had a 4.3 (95 % CI 3.1–6.0), 18.0 (95 % CI 13.3–24.5), and 58.5 (95 % CI 42.9–79.9) times increased risk of metabolic syndrome compared with patients in Q1 (Table 3). In addition, WHtR quartiles had highest odd ratios for metabolic syndrome among BMI, FM index, TFM index, and WHtR in both Model 1 and Model 2.

**Table 3:** Odds ratios for metabolic syndrome according to the quartiles of variable indices

	Q1	Q2	Q3	Q4
Males				
Model 1				
BMI	–	3.0 (2.4–3.7)	6.0 (5.0–7.4)	17.3 (14.2–21.0)
FM index	–	3.7 (3.0–4.6)	7.9 (6.4–9.7)	16.5 (13.5–20.3)
TFM index	–	2.2 (1.8–4.8)	4.0 (3.4–4.8)	9.6 (8.1–11.5)
WHtR	–	4.4 (3.4–5.7)	11.5 (9.0–14.7)	44.3 (34.6–56.7)
Model 2				
BMI	–	3.5 (2.8–4.3)	7.7 (6.2–9.5)	28.3 (22.7–35.1)
FM index	–	3.8 (3.0–4.7)	8.1 (6.6–10.0)	20.0 (16.1–24.8)
TFM index	–	2.5 (2.1–3.1)	4.7 (3.9–5.7)	12.3 (10.2–14.9)
WHtR	–	4.0 (3.1–5.2)	9.6 (7.5–12.3)	36.1 (28.0–46.4)
Females				
Model 1				
BMI	–	3.8 (3.1–4.7)	9.9 (8.2–12.0)	27.1 (22.3–32.8)
FM index	–	3.2 (2.7–3.9)	7.7 (6.5–9.2)	18.7 (15.6–22.2)
TFM index	–	2.6 (2.2–3.1)	5.5 (4.7–6.5)	14.9 (12.7–17.5)
WHtR	–	6.2 (4.6–8.5)	33.0 (24.5–44.4)	129.3 (95.7–174.6)
Model 2				
BMI	–	3.4 (2.7–4.2)	8.3 (6.7–10.2)	25.8 (20.8–32.0)
FM index	–	3.5 (2.8–4.3)	7.0 (5.8–8.6)	18.4 (15.0–22.4)
TFM index	–	2.2 (1.8–2.6)	4.1 (3.4–4.9)	12.4 (10.3–14.9)
WHtR	–	4.3 (3.1–6.0)	18.0 (13.3–24.5)	58.5 (42.9–79.9)

Model 1 was unadjusted, whereas model 2 was adjusted for age, mean daily alcohol intake, smoking, and physical activity. Variables were expressed as odds ratio (95 % confidence interval), and odds ratios were calculated for Q1

*Abbreviations*: *BMI* body mass index; *FM* fat mass; *TFM* trunk fat mass; *WHtR* waist to height ratio; *CI* confidence interval; *Q1* first quartile; *Q2* second quartile; *Q3* third quartile; *Q4* fourth quartile

Statistical significance was defined as *P* < 0.001 for all analyses

The patients had more metabolic syndrome components on both univariate and multivariate analyses as the WHtR quartiles increased (Table 4). In addition, the WHtR quartiles had the highest odds ratios for predicting all metabolic syndrome components except elevated blood pressure in males (Additional file 1: Table S3). Logistic regression analyses by using a cut-off value of WHtR < 0.5 as an international guideline showed a significant association between WHtR < 0.5 and metabolic syndrome or metabolic syndrome components (Additional file 1: Table S4).

**Table 4:** Number of metabolic syndrome components by the WHtR quartile

	Q1	Q2	Q3	Q4
Males				
Model 1				
Mean (95 % CI)*	0.711 (0.657–0.765)	1.369 (1.315–1.423)	2.050 (1.996–2.104)	3.067 (3.013–3.121)
Model 2				
Mean*	0.830 (0.774–0.885)	1.387 (1.334–1.441)	1.998 (1.945–2.052)	2.997 (2.923–3.032)
Model 3				
Mean*	0.883 (0.780–0.985)	1.406 (1.295–1.518)	2.010 (1.894–2.125)	3.014 (2.906–3.123)
Females				
Model 1				
Mean (95 % CI)*	0.538 (0.494–0.581)	1.176 (1.132–1.220)	2.335 (2.291–2.379)	3.288 (3.244–3.331)
Model 2				
Mean*	0.840 (0.794–0.885)	1.255 (1.214–1.297)	2.220 (2.178–2.262)	3.011 (2.966–3.056)
Model 3				
Mean*	0.739 (0.603–0.876)	1.154 (1.016–1.291)	2.156 (2.007–2.304)	2.921 (2.777–3.065)

Model 1 was unadjusted, whereas model 2 was adjusted for age, mean daily alcohol intake, smoking, and physical activity. Model 3 was adjusted for age, mean daily alcohol intake, smoking, and physical activity and accounted for the complex sampling design and appropriate sampling weight of the national survey

*Abbreviations*: *WHtR* waist to height ratio; *CI* confidence interval; *Q1* first quartile; *Q2* second quartile; *Q3* third quartile; *Q4* fourth quartile

*Mean (95 % CI) were calculated by using analysis of covariance and statistical significance was defined as *P* < 0.001 for all analyses

## Discussion

The present study demonstrates that WHtR had the highest AUC and was the best predictor of metabolic syndrome and components of metabolic syndrome for both sexes. Univariate and multivariate analyses revealed that WHtR quartiles were associated with metabolic syndrome in the general population. In addition, the WHtR quartiles had the highest odds ratio for predicting most metabolic syndrome components (elevated fasting glucose, blood pressure, triglyceride level, and decreased high-density lipoprotein level) among the other indices in both sexes. Logistic regression analyses with a cut-off value of WHtR < 0.5 as an international guideline showed a significant association with metabolic syndrome or metabolic syndrome components.

Obesity is a well-known risk factor for and component of metabolic syndrome. Central visceral fat has shown stronger associations with cardiovascular disease risk and metabolic syndrome than subcutaneous fat [25, 26]. Previous studies have demonstrated that WC is closely correlated to reference methods such as computed tomography or magnetic resonance imaging and is a more predictive surrogate for metabolic syndrome than BMI [6, 7, 9, 27, 28]. Height is inversely associated with cholesterol level, and WC would be needed to adjust for height [29]. Therefore, WHtR may be a more predictive measure than classic body indices such as WC and BMI. A meta-analysis showed the superiority of WHtR [9].

The present study compared the anthropometric (WHtR and BMI) and body composition indices. Our results shows that body indices using a body composition analyzer have no advantage over classic anthropometric indices. Our results showed that WHtR had the highest AUC for predicting metabolic syndrome among other body indices including BMI, FM, LM, SM, BMC, and TFM for both sexes. Logistic regression analyses showed that the WHtR quartiles had the highest odds ratio for predicting metabolic syndrome or each component among the BMI, FM index, TFM index, and WHtR. There was a significant difference in numbers of metabolic syndrome components using the general linear model and these data compensate for the weakness of the dichromatic category for metabolic syndrome. Our results show that body indices measured using the body composition analyzer have no advantage over classic anthropometric indices. Zhang et al. enrolled Asians and showed that the sensitivity and specificity of WHtR were 75.9 % and 52.7 % in males and 67.8 % and 62.5 % in females, respectively [18]. The values in their study were lower than those in the present study, while the cut-off values in their study were similar to those in the present study.

Classic body indices cannot distinguish between fat and other components. Body composition analyzers such as bioimpedance analysis and DEXA, measure FM and free-fat mass, which include BMC and LM. These indices may be a more optimal marker for prediction of metabolic syndrome or insulin resistance than classic indices. Kim et al. showed that the FMI evaluated with bioimpedance analysis was the most strongest predictor of metabolic syndrome among Korean men [14]. Namwongprom et al. enrolled Thai adults and showed that the android-to-gynoid FM ratio calculated by using DEXA was more predictive in the diagnosis of metabolic syndrome than either the WC or BMI [15]. A previous study in a Turkish population showed that the WHtR is the most predictive indicator among the anthropometric indices; however, visceral fat measured with bioimpedance analysis is a more sensitive variable than the WHtR [16]. However, many studies did not show stronger associations between body composition indices and metabolic syndrome and/or components of metabolic syndrome than classic indices [18, 30–34]. Bioimpedance analysis or DEXA can measure the exact FM. Although FM is associated with development of metabolic syndrome, there have been controversies regarding the advantage of FM in prediction of metabolic syndrome. Many studies have shown that FM has no advantage over classic body indices [18, 30–32]. Some recent studies enrolled migrant Asian Indian, Chinese, or multiethnics participants and compared various anthropometric indices with indices measured with DEXA [17–19]. However, those studies did not show a superiority of the indicators measured with body composition analyzers over either the WC or WHtR. Bosy-Westphal et al. suggested that the inaccuracies of impedance measurement may be associated with discrepancies among studies [35]. DEXA is a reference method for measurement of FM. However, simple trunk or total FM cannot differentiate between subcutaneous and visceral fat. In addition, previous studies have shown that an increase in LM may be associated with an increase in the prevalence of metabolic syndrome. LM plays an important role in maintenance of systemic glucose metabolism [36]. Many clinicians believe that LM may be a protective effect of development of metabolic syndrome. Previous studies have shown that an increase in LM is positively associated with the risk of developing insulin resistance and metabolic syndrome [29, 33]. Although the exact mechanism for positive correlation remains poorly understood, its association may be linked to a paradoxical decrease in LM to metabolize glucose for energy as LM increases [37]. These findings demonstrate that indices using body composition analyzers have no advantage over classic body indices.

A systematic review of many prospective or cross-sectional studies and involving many ethnic groups suggested that 0.5 is an appropriate boundary value for predicting cardiovascular disease and diabetes mellitus in both sexes [38]. Therefore, clinical practitioners simply recommend that “your waist circumference should be less than half your height” [19]. The review with 147 individual analyses showed that the mean AUROC values for predicting metabolic syndrome were approximately 0.704, 0.693, and 0.670 for the WHtR, WC, and BMI, respectively. The review showed that the WHtR had the highest AUROC value followed by the WC, and then the BMI; however, the difference in the AUROC was small. The AUROC value for predicting metabolic syndrome, as well as the differences in AUROC between the WHtR and other indices, was greater in our study than in the previous review. In addition, our cut-off value was similar to that of the review. Therefore, the criteria for other population groups are applicable to the Korean population. In our data, logistic regression analyses by using a cut-off value of WHtR < 0.5 showed a significant association between WHtR < 0.5 and metabolic syndrome or metabolic syndrome components.

The present study was based on a sub-sample of a nationwide representative survey. Approximately 57.7 % of the total cohort were excluded from the study cohort. Additional file 1: Table S1 shows the baseline characteristics of the total cohort. Age and education level were lower in the total cohort than in the study cohort. Concerning the mean daily alcohol intake, a higher abstinence and lower heavy drinking was found in the total cohort than in the study cohort. The number of non-smokers was higher in the total cohort than in the study cohort. The amount of exercise was similar between the two cohorts. The age of participants was lower in the total cohort than in the study cohort. The participants’ age may be associated with the differences in alcohol drinking, education level, and smoking status. The total cohort included 8,995 participants who were younger than 18 years. This exclusion may have caused older age in the study cohort than in the total cohort.

The present study has a number of limitations. First, it is limited by its cross-sectional nature and ethnic differences were not evaluated. This study design showed associations between the indices and metabolic diseases but could not establish the causality between the indices and metabolic syndrome, and the ability of the indices to predict metabolic diseases. Second, the present study has the probability of recall bias from a self-reporting questionnaire survey. The impact of these limitations is reduced by the large sample size in the present study, but did not offset the cross-sectional nature of the study. A prospective study including follow-up data is needed to show the cause-and-effect relations between indices and metabolic diseases.

## Conclusion

Body indices using body composition analyzers have no advantage over classic body indices such as BMI and WHtR. Among the classic body indices, WHtR is most useful to predict the presence of metabolic syndrome in the Korean population. In addition, it is really cheap and safe, especially if compared with DEXA.

## Additional file

Additional file 1: Table S1. Baseline characteristics of the total cohort. **Table S2.** Comparison of AUROC for prediction of metabolic syndrome among variable indices. **Table S3.** Odds ratios for metabolic syndrome components according to the quartiles of variable indices. **Table S4.** Odds ratios for metabolic syndrome components according to the quartiles of variable indices. (DOCX 29 kb)

## Abbreviations

WC: Waist circumference
BMI: Body mass index
WHtR: Waist to height ratio
DEXA: Dual-energy X-ray absorptiometry
FM: Fat mass
KNHANES: Korean National Health and Nutrition Examination Survey
LM: Lean mass
TFM: Trunk fat mass
BMC: Bone mineral content
eGFR: Estimated glomerular filtration rate
MET min/wk: Metabolic equivalent-minutes per week
PYR: Average daily smoking amount [pack] × smoking period [year]
CI: Confidence interval
AUROC: Area under the receiver operating characteristic curve
Q1: First quartile
Q2: Second quartile
Q3: Third quartile
Q4: Fourth quartile
