# Supplementary material for: Comparison of waist to height ratio and body indices for prediction of metabolic disturbances in the Korean population: the Korean National Health and Nutrition Examination Survey 2008–2011
Source: BMC Endocr Disord. 2015 Dec 8;15:79. doi: 10.1186/s12902-015-0075-5 (PMC4672527; doi:10.1186/s12902-015-0075-5)
Supplement: Additional file 1: Table S1. — Baseline characteristics of the total cohort. Table S2. Comparison of AUROC for prediction of metabolic syndrome among variable indices. Table S3. Odds ratios for metabolic syndrome components according to the quartiles of variable indices. Table S4. Odds ratios for metabolic syndrome components according to the quartiles of variable indices. (DOCX 29 kb) [file 12902_2015_75_MOESM1_ESM.docx]

**Table S1. Baseline characteristics of the total cohort**

| **Characteristics** | **Males (n = 17,195)** | **Females (n = 20,558)** | ***P* value*** |
| --- | --- | --- | --- |
| Age | 38 (16**–57**) | 41 (23**–59**) | <0.001 |
| Education level |  |  | <0.001 |
| Up to elementary school | 6063 (35.3%) | 8729 (42.5%) |  |
| Middle school | 2027 (11.8%) | 2130 (10.4%) |  |
| High school | 4211 (24.5%) | 4967 (24.2%) |  |
| College or higher | 3679 (21.4%) | 3655 (17.8%) |  |
| No data | 1215 (7.1%) | 1077 (5.2%) |  |
| Smoking |  |  | <0.001 |
| Heavy smoker | 1307 (7.6%) | 52 (0.3%) |  |
| Intermediate smoker | 5843 (34.0%) | 1350 (6.6%) |  |
| Ex-smoker | 2111 (12.3%) | 405 (2.0%) |  |
| Non-smoker | 5489 (31.9%) | 16519 (80.4%) |  |
| No data | 2445 (14.2%) | 2232 (10.9%) |  |
| Mean daily alcohol intake |  |  | <0.001 |
| Abstinence | 5234 (30.4%) | 8923 (43.4%) |  |
| Moderate drinking | 9035 (52.5%) | 9289 (45.2%) |  |
| Heavy drinking | 855 (5.0%) | 419 (2.0%) |  |
| No data | 2071 (12.0%) | 1927 (9.4%) |  |
| Exercise |  |  | <0.001 |
| ≥6000 MET min/wk | 2914 (16.9%) | 2385 (11.6%) |  |
| 3000~6000 MET min/wk | 2771 (16.1%) | 2630 (12.8%) |  |
| <3000 MET min/wk | 6320 (36.8%) | 9824 (47.8%) |  |
| No data | 5190 (30.2%) | 5719 (27.8%) |  |

Data are expressed as numbers (percentages) for categorical variables and mean ± standard deviation for continuous variables.

*Statistical significance was tested the Mann-Whitney U-test for continuous variables and Pearson χ^2^ test or Fisher exact test for categorical variables.

Abbreviations: MET min/wk, metabolic equivalent-minutes per week.

**Table S2. Comparison of AUROC for prediction of metabolic syndrome among variable indices**

| **Variables** | **AUC (95% CI)** | **Cutoff value** | **Sensitivity** | **Specificity** | ***P* value*** |
| --- | --- | --- | --- | --- | --- |
| Males |  |  |  |  |  |
| BMI | 0.763 (0.753-0.773) | >23.74 | 78.8% | 60.5% | <0.001 |
| FM index | 0.754 (0.744-0.764) | >5.31 | 75.6% | 62.6% | <0.001 |
| LM index | 0.685 (0.674-0.696) | >17.78 | 60.7% | 66.5% | <0.001 |
| BMC index | 0.573 (0.561-0.585) | >0.87 | 63.4% | 48.0% | <0.001 |
| SM index | 0.611 (0.600-0.623) | >7.78 | 55.2% | 61.6% | <0.001 |
| TFM index | 0.727 (0.717-0.738) | >8.78 | 69.6% | 63.9% | <0.001 |
| WHtR | 0.823 (0.814-0.832) | >0.51 | 74.0% | 75.8% | - |
| Females |  |  |  |  |  |
| BMI | 0.795 (0.786-0.802) | >23.59 | 74.9% | 70.6% | <0.001 |
| FM index | 0.769 (0.760-0.777) | >7.96 | 71.4% | 69.8% | <0.001 |
| LM index | 0.749 (0.740-0.758) | >14.68 | 70.1% | 67.2% | <0.001 |
| BMC index | 0.574 (0.564-0.584) | ≤0.75 | 45.1% | 70.0% | <0.001 |
| SM index | 0.682 (0.672-0.691) | >5.80 | 70.1% | 56.5% | <0.001 |
| TFM index | 0.761 (0.751-0.769) | >7.70 | 68.4% | 70.3% | <0.001 |
| WHtR | 0.870 (0.863-0.877) | >0.51 | 83.3% | 76.5% | - |

*The calculated statistical significance between the WHtR and other indices.

Abbreviations: AUROC, area under the receiver operating characteristic curve; AUC, area under the curve; CI, confidence interval; BMI, body mass index; FM, fat mass; LM, lean mass; BMC, bone mineral content; SM, skeletal muscle mass; TFM, trunk fat mass; WHtR, waist to height ratio.

**Table S3. Odds ratios for metabolic syndrome components according to the quartiles of variable indices**

|  | **Q1** | **Q2** | **Q3** | **Q4** |
| --- | --- | --- | --- | --- |
| **Males** |  |  |  |  |
| Elevated FG |  |  |  |  |
| BMI | **–** | **1.8 (1.5–2.1)** | 2.6 **(2.2–3.1)** | 3.8 **(3.2–4.5)** |
| FM index | **–** | **1.9 (1.6–2.2)** | **2.4 (2.1–2.9)** | **3.3 (2.8–3.9)** |
| TFM index | **–** | **1.7 (1.4–1.9)** | **2.2 (1.9–2.6)** | **3.6 (3.1–4.2)** |
| WHtR | **–** | **1.9 (1.6–2.3)** | **3.0 (2.5–3.5)** | **4.1 (3.5–4.9)** |
| Elevated BP |  |  |  |  |
| BMI | **–** | **1.9 (1.6–2.2)** | **2.5 (2.2–3.0)** | **4.3 (3.7–5.1)** |
| FM index | **–** | **1.7 (1.5–2.0)** | **2.4 (2.0–2.8)** | **3.6 (3.1–4.2)** |
| TFM index | **–** | **1.6 (1.4–1.9)** | **2.3 (2.0–2.7)** | **3.3 (2.9–3.9)** |
| WHtR | **–** | **1.7 (1.4–2.0)** | **2.1 (1.8–2.4)** | **3.7 (3.1–4.3)** |
| Elevated TG |  |  |  |  |
| BMI | **–** | **2.2 (1.9–2.6)** | **3.4 (2.9–4.0)** | **5.6 (4.8–6.6)** |
| FM index | **–** | **3.0 (2.6–3.6)** | **4.5 (3.8–5.3)** | **6.5 (5.5–7.7)** |
| TFM index | **–** | **1.6 (1.4–1.9)** | **2.4 (2.0–2.8)** | **3.3 (2.9–3.8)** |
| WHtR | **–** | **3.0 (2.6–3.6)** | **5.2 (4.4–6.2)** | **7.4 (6.2–8.8)** |
| Decreased HDL-C |  |  |  |  |
| BMI | **–** | **2.2 (1.9–2.6)** | **3.0 (2.6–3.6)** | **4.6 (3.9–5.4)** |
| FM index | **–** | **2.2 (1.9–2.6)** | **3.2 (2.7–3.8)** | **4.4 (3.8–5.2)** |
| TFM index | **–** | **1.7 (1.5–2.0)** | **2.2 (1.9–2.6)** | **3.4 (2.9–4.0)** |
| WHtR | **–** | **2.3 (1.9–2.7)** | **3.4 (2.9–4.0)** | **4.7 (4.0–5.6)** |
| **Females** |  |  |  |  |
| Elevated FG |  |  |  |  |
| BMI | **–** | **1.6 (1.3–1.9)** | **2.3 (2.0–2.8)** | **4.3 (3.7–5.1)** |
| FM index | **–** | **1.7 (1.5–2.1)** | **2.2 (1.9–2.6)** | **3.7 (3.1–4.3)** |
| TFM index | **–** | **1.5 (1.3–1.8)** | **2.2 (1.9–2.7)** | **4.3 (3.6–5.0)** |
| WHtR | **–** | **2.0 (1.6–2.4)** | **3.1 (2.6–3.8)** | **5.8 (4.7–7.0)** |
| Elevated BP |  |  |  |  |
| BMI | **–** | **1.6 (1.4–1.9)** | **2.2 (1.9–2.7)** | **4.6 (3.9–5.5)** |
| FM index | **–** | **1.4 (1.2–1.7)** | **2.1 (1.8–2.5)** | **3.6 (3.0–4.2)** |
| TFM index | **–** | **1.3 (1.1–1.5)** | **2.0 (1.7–2.3)** | **3.6 (3.0–4.2)** |
| WHtR | **–** | **1.8 (1.5–2.2)** | **3.0 (2.4–3.6)** | **4.8 (4.0–5.9)** |
| Elevated TG |  |  |  |  |
| BMI | **–** | **1.8 (1.5–2.2)** | **3.1 (2.6–3.7)** | **4.6 (3.8–5.4)** |
| FM index | **–** | **2.5 (2.1–3.0)** | **3.4 (2.8–4.0)** | **5.0 (4.2–5.9)** |
| TFM index | **–** | **1.3 (1.1–1.6)** | **2.0 (1.7–2.3)** | **3.2 (2.7–3.8)** |
| WHtR | **–** | **2.8 (2.3–3.5)** | **4.6 (3.8–5.7)** | **7.8 (6.3–9.6)** |
| Decreased HDL-C |  |  |  |  |
| BMI | **–** | **1.5 (1.3–1.6)** | **2.0 (1.7–2.2)** | **2.8 (2.5–3.2)** |
| FM index | **–** | **1.6 (1.4–1.8)** | **2.0 (1.8–2.3)** | **2.6 (2.3–3.0)** |
| TFM index | **–** | **1.2 (1.1–1.4)** | **1.5 (1.3–1.7)** | **2.4 (2.1–2.7)** |
| WHtR | **–** | **1.9 (1.6–2.1)** | **2.5 (2.2–2.9)** | **3.9 (3.4–4.6)** |

Variables are expressed as odds ratio (95% confidence interval), and odds ratio were calculated for Q1.

Odds ratios were calculated by using multivariate logistic regression models, and statistical significance was defined as *P* < 0.05 for all analyses. The model was adjusted for age, mean daily alcohol intake, smoking, and physical activity.

Abbreviations: FG, fasting glucose; BP, blood pressure; TG, triglyceride; HDL-C, high-density lipoprotein cholesterol; BMI, body mass index; FM, fat mass; TFM, trunk fat mass; WHtR, waist to height ratio; Q1, first quartile; Q2, second quartile; Q3, third quartile; Q4, fourth quartile.

**Table S4. Odds ratios for metabolic syndrome components according to the quartiles of variable indices**

|  | **Metabolic syndrome** | **Elevated FG** | **Elevated BP** | **Elevated TG** | **Decreased HDL-C** |
| --- | --- | --- | --- | --- | --- |
| **Model 1** |  |  |  |  |  |
| **Males** | **8.5 (7.5–9.6)** | **3.1 (2.8–3.4)** | **2.8 (2.6–3.1)** | **3.1 (2.8–3.5)** | **2.5 (2.3–2.8)** |
| **Females** | **18.1 (15.9–20.6)** | **4.4 (3.9–4.9)** | **6.1 (5.5–6.7)** | **4.7 (4.2–5.3)** | **2.8 (2.6–3.0)** |
| **Model 2** |  |  |  |  |  |
| **Males** | **7.3 (6.4–8.2)** | **2.4 (2.1–2.7)** | **2.1 (1.8–2.3)** | **3.1 (2.8–3.5)** | **2.4 (2.1–2.7)** |
| **Females** | **10.5 (9.2–12.1)** | **2.6 (2.3–3.0)** | **2.5 (2.2–2.8)** | **2.9 (2.6–3.3)** | **2.1 (1.9–2.3)** |

Variables are expressed as odds ratio (95% confidence interval).

Model 1 was unadjusted, whereas model 2 was adjusted for age, mean daily alcohol intake, smoking, and physical activity. Variables are expressed as odds ratio (95% confidence interval), and odds ratio were calculated for WHtR < 0.5.

Statistical significance was defined as *P* < 0.001 for all analyses.

Abbreviations: FG, fasting glucose; BP, blood pressure; TG, triglyceride; HDL-C, high density lipoprotein cholesterol.
